# Response to single dose hepatitis B vaccine in Congolese non-HIV hemodialysis patients: a prospective observational study

**DOI:** 10.11604/pamj.2019.34.122.19603

**Published:** 2019-10-31

**Authors:** Pitchou Yemasai Kengibe, Jean-Robert Risassy Makulo, Yannick Mayamba Nlandu, François Bompeka Lepira, Ernest Kiswaya Sumaili, Justine Busanga Bukabau, Charles Nlombi Mbendi, Steve Mundeke Ahuka, Antoine Wola Tshimpi, Patrick Kisoko Ngoma, Nseka Nazaire Mangani, Sebastien Nsukini Mbendi

**Affiliations:** 1 Division of Hepato-gastroenterology, University of Kinshasa, Kinshasa, Democratic Republic of the Congo; 2 Division of Nephrology, University of Kinshasa, Kinshasa, Democratic Republic of the Congo; 3 Hemodialysis Unit of Ngaliema Medical Center, Kinshasa, Democratic Republic of the Congo; 4 Division of Microbiology, University of Kinshasa, Kinshasa, Democratic Republic of the Congo; 5 Climy Medical Center of Hepato-gastroenterology, Kinshasa, Democratic Republic of the Congo

**Keywords:** Hemodialysis, hepatitis B virus, vaccination, antibodies seroconversion

## Abstract

**Introduction:**

Because of the cost, in the hemodialysis centers of Kinshasa, the double dose of hepatitis B (HBV) vaccine is administered only to HIV infected patients while other patients receive a single dose. This study aimed to evaluate the single-dose vaccination Protocol and identify determinants of seroconversion's lack of anti-HBs after vaccination schedule.

**Methods:**

56 non-HIV chronic hemodialysis patients serologically negative for HBs Ag, anti-HBs and anti-HBc were selected between January 2014 and December 2016. The recombinant DNA vaccine (Euvax B^®^20 μg) was administered intramuscularly in the deltoid muscle at days 0, 30, 60 and 180. Serum anti-HBs titer was assayed at day 240. The endpoint was seroconversion, defined as anti-HBs titer ≥ 10 IU/l (10-99 IU/l = low protective vaccine response; ≥ 100 IU/l = highly protective vaccine response). Anti-HBs titer < 10 IU/l defined a lack of seroconversion. A Logistic regression model was used to identify factors associated with the lack of seroconversion.

**Results:**

In the study group (mean age 55.6± 15.1 years; 73 % men, 36% diabetic and 86% hypertensive), low and highly protective vaccine responses were seen in 32% and 50% respectively versus 18% of patient had a lack of seroconversion. CRP > 6 mg/L (aOR: 8.96), hypoalbuminemia (aOR: 6.50) and KT/V < 1.2 (aOR: 3.70) were associated with the lack of seroconversion.

**Conclusion:**

Half of the patients in the study had either a lack or low protective vaccine response. Patient-related factors and hemodialysis parameters were the main factors associated with the lack of anti-HbS seroconversion. These results highlight the need to maximize doses of vaccine in all patients.

## Introduction

Hepatitis B virus (HBV) is relatively stable in the environment and remains viable for at least one week on environmental surfaces at room temperature; its transmission happens through per cutaneous or mucosal exposition to infected blood or body fluids [1]. It is the most causes of cirrhosis and hepatocellular carcinoma in the world [2]. The prevalence of this infection varies worldwide. Higher prevalence is encountered in low-income countries, including those of sub-Saharan Africa (SSA) where HBV infection is hyperendemic. Indeed, in the general population, more than 8% of people are hepatitis B surface antigen (HBs Ag) chronic carriers [3]. Five HBV genotypes are more frequently detected in Africa, A, B, C, D and E genotypes [4]. Patients on chronic hemodialysis are considered as a high risk group for hepatitis B infection because of many therapeutic procedures routinely used in this group increase probability of HBV infection [5]. In this regard, HBs Ag has been detected in dialysis centers on clamps, scissors, dialysis machine control knobs, and door knobs [6]. Thus, blood-contaminated surfaces that are not routinely cleaned and disinfected represent a reservoir for HBV transmission. In addition, dialysis staff members can transmit HBV to patients from contaminated surfaces by hands, gloves or through use of contaminated equipment and supplies [6]. Thus, controlling the spread of HBV infection in dialysis centers has been one of the major advances in the treatment of patients with end stage renal disease (ESRD). Nowadays, especially in developed countries, the prevalence of HBs Ag carriers on chronic dialysis has decreased significantly through preventive measures, such as routine vaccination of patients and health care workers, regular use of erythropoietin as a substitute for blood transfusions, early serological diagnosis, isolation of infected patients, and cleaning and disinfecting procedures [7]. Many protocols recommend routine HBV vaccination prior to dialysis with four doses of 40 μg each administered intramuscularly in the deltoid muscle within 0, 1, 2, and 6 months of the procedure [7-9]. However several worldwide studies have shown that one-third of chronic hemodialysis patients do not respond adequately to immunization [7, 10, 11]. The decline in immunity observed in chronic hemodialysis patients plays an important role in explaining this non-response [12]. Few studies have examined the efficacy of HBV vaccination in SSA chronic hemodialysis patients [13, 14]. Several specificities can influence the HBV vaccine response in this population. Indeed chronic kidney disease is usually detected very late, at stage requiring dialysis in emergency; in addition, patients generally have many comorbidities and present uremic syndrome that may reduce their immunity [15]. More often, patients who have to support themselves their medical care cannot afford the cost of double dose of HBV vaccine. Therefore, in DR Congo, many centers usually provide a single-dose regimen to HD patients, unless they have HIV to receive the double dose. The present study aimed to evaluate the single-dose vaccination's Protocol and identify determinants of the lack of seroconversion in chronic hemodialysis patients.

## Methods

We conducted a prospective study including all consent patients who started chronic hemodialysis treatment in three centers in Kinshasa, DR Congo (Division of Nephrology, University of Kinshasa; Hemodialysis Unit of Ngaliema Medical Center, Kinshasa, and Afia Medical Center of Hemodialysis, Kinshasa) during the period from January 1^st^ 2014 to December 31^st^ 2016. Only patients who were serologically negative for HBs Ag, anti-HBs and antibody against hepatitis B core antigen (anti-HBc) were selected. The diagnosis of End stage renal disease (ESRD) and the indication of chronic hemodialysis treatment were made according to the 2012 KDIGO's recommendations [16]. The vaccines Euvax^®^ used were administered intramuscularly to the deltoid muscle in single dose (20 μg each) at days 0, 30, 60 and 180. Patients who received a double dose of vaccine were not included in the present analyzes (HIV-infected patients and some patients infected with the hepatitis C virus). Serum was collected at day 240 to perform quantitative tests, measuring the anti-HBs titer. The endpoint was seroconversion defined as anti-HBs titer ≥ 10 IU/l (10-99 IU/l=low protective vaccine response; ≥ 100 IU/l = highly protective vaccine response) while anti-HBs titer < 10 IU/l defined the lack of seroconversion [7, 17]. Other parameters of interest studied were as follows: etiology of chronic kidney disease (CKD), co-morbidities, residual diuresis (RD), C Reactive protein (CRP), serum albumin, tobacco use, alcohol consumption, number of dialysis sessions per week and KT/V.

**Statistical analysis:** data were analyzed using SPSS software version 21. Tables or graphs were used for the presentation of results. Quantitative variables were presented as mean ± standard deviation (SD) and qualitative variables as a percentage. Comparison of proportions, medians and averages was performed using Chi-square or Fisher exact tests, Mann Whitney Wilcoxon and Student t-tests, respectively. A Logistic regression model was used to identify factors associated with the lack of anti-HBs seroconversion. A value of p <0.05 defined the threshold of statistical significance.

**Ethical Considerations:** this study has been approved by the Ethics Committee of the School of Public Health of the University of Kinshasa, referenced ESP/CE/013/2017. Patients' recruitment was done anonymously and on the basis of free and informed consent in accordance with Helsinki's recommendations.

## Results

**General characteristics:** all three hemodialysis centers used Fresenius generators and high flux permeability dialyzers. A total of 117 chronic hemodialysis patients were recruited, however 26 did not meet the inclusion criteria. During the study, 13 patients were deceased, 10 lost to follow-up, 8 traveled abroad and 4 patients were non-compliant to the vaccination schedule. Thus, 56 patients were included in the present analysis (73% men). Their mean age was 55.6 ± 15.1 years and 36% of them were diabetic versus 86% hypertensive.

**The vaccine response:** in the whole group, the lack of vaccine response was encountered in 18% versus 82% of patients had seroconvesion (a low protective response and a highly protective in 32% and 50%, respectively). Age, body mass index (BMI), hypertension as well as albuminemia, CRP and KT/V showed a difference between groups according to the vaccine response (Table 1, Table 2).

**Table 1:** Clinical and biological parameters as function of the HBV vaccine response

Variable	Seroconversion n=46	Lack n=10	P
Men	34 (73.9)	7 (70.0)	0.539
Age, years	60 (51-66)	49 (38-70)	0.727
Hypertension, %	38 (82.6)	8 (80.0)	0.184
Diabetes, %	17 (37.0)	3 (30.0)	0.839
History of transfusion	27 (58.7)	8 (80.0)	0.186
Alcohol, %	24 (52.2)	7 (70.0)	0.252
Tobacco, %	13 (28.2)	3 (30.0)	0.594
RD ≤ 500 ml/day, %	25 (54.3)	5 (50.0)	0.537
BMI, Kg/m2	25 (23-27)	22 (21-33)	0.727
Albuminemia, g/L	32 (26-38)	16 (11-19)	0.001
CRP, mg/L	7 (4-14)	15 (9-24)	0.015
Hb, g/dL	10.0 (8.6-11.4)	9.8 (7.3-11.2)	0.424

BMI: body mass index; CRP: C reactive protein; RD: residual diuresis; record values are expressed as absolute frequency (%) or median (IQR 25–75)

**Table 2:** Hemodialysis parameters as function of the HBV vaccine response

Hemodialysis parameters	Seroconversion n=46	Lack n=10	P
Vascularaccess			0.315
Catheter	37 (80.4)	7 (70.0)	
Fistula	9 (20.6)	3 (30.0)	
KT/V < 1.2	3.5 (2.4-3.7)	1.7 (1.2-2.0)	0.005
Number of sessions			0.274
≤ 2/week	20 (43.5)	6 (60.0)	
3/week	26 (56.5)	4 (40.0)	

Record values are expressed as absolute frequency (%) or median (IQR 25–75)

**Risk factors of the lack of anti-HbS seroconversion:** CRP > 6 mg/L, albuminemia < 30 g/L and KT/V < 1.2 have emerged as the main factors associated with the lack of the anti-HbS seroconversion (Table 3).

**Table 3:** Risk factors of the lack of Anti-HbS seroconversion after single vaccine schedule

	p	OR	CI 95%
Albuminemia< 30 g/L	< 0.001	1.59	1.19-2.12
KT/V < 1.2	< 0.001	26.70	4.54 - 156.75
CRP > 6 mg/dL	0.001	1.50	1.17 - 1.93

## Discussion

Our finding of a low HBV vaccine response is consistent with previous reports on HBV vaccine among chronic HD patients relative to the general population. Indeed, while HBV vaccination induces seroprotection in 90 to 95% of cases in the general population, immunogenicity is lower in patients with renal failure [7, 10, 11]. This weak induced immunogenicity is thought to rely upon an altered humoral and cellular immunity with subsequent decrease in the activity of immune system cells (B and T lymphocytes, monocytes, macrophages) [12, 13]. This decreased cellular activity results in reduced phagocytosis by polynuclear cells, production of interleukin-1 and interleukin-2 by macrophages and T4 lymphocytes, respectively, and production of antibodies by B lymphocytes [13]. Potential factors explaining this altered immune system activity include, among others, decreased uremic toxin clearance, nutritional deficiency and, also, immunosuppressive drugs used to treat some glomerular diseases [13, 14]. This immune dysfunction is responsible for the decline in the vaccine seroconversion rate as well as a more rapid decrease in antibody levels compared to healthy subjects [7, 10, 11].

The anti-HbS seroconversion rate in our series is similar to that reported by Boumansour *et al*. in Algeria and Feriani *et al*. in Morocco, who reported a seroconversion rate of 76% and 85%, respectively [18, 19]. Ibrahim *et al*. in Egypt reported 93% of seroconversion [20]. However Ayub *et al*. reported 59% of seroconversion in Brazil [21]. Studies in Europe and North America have also shown divergent results [22-24]. It is difficult to compare results of different studies because of differences in criteria of selection and methodology used. Multivariate analysis showed that several factors could influence the vaccine response in hemodialysis patients. The chronic inflammatory state, as expressed by CRP levels, had a negative influence on the vaccine response. Our findings are consistent with those of other authors who noted a decrease in CRP in patients performing dialysis with high-throughput membranes and consequently a better vaccine response compared with patients with a marked inflammatory state using low-flow membrane [25]. Chronic inflammation is associated with an immune deficiency that explains the decline in vaccine response in this population. The mechanisms responsible for this chronic inflammatory state in the dialysis, as reviewed in details by Kaysen [26], are multiple and include those secondary to CKD such as oxidative stress due to the decrease in anti-oxidative defenses and the accumulation of toxic molecules such as advanced glycation end-products [26]. Inflammatory phenomenon induced by hemodialysis treatment may be linked to the interaction between the blood and the hemodialysis membrane or to the quality of the water used for HD [26]. Schiffl *et al*. have shown that the use of an ultrapure dialysate reduces the average serum levels of CRP in hemodialysis patients [25].

Hypoalbuminemia showed a negative impact on the vaccine response to HBV. Malnutrition, with subsequent hypoalbuminemia, has a negative influence on the vaccine response. This condition is common in hemodialysis patients and can be explained by loss of appetite due to uremic waste products and toxins, intermediate metabolic abnormality and increased catabolism due to hyperparathyroidism and acidosis, loss of amino acids and glucose in dialysate and chronic inflammation [27]. KT/V <1.2 was significantly associated with lack of response. Many studies have shown association between vaccine response to HBV and the quality of dialysis expressed by KT/V [28]. In our study, age did not influence seroconversion, perhaps because of the small sample. Previous reports from the literature found a declining vaccine response with increasing age [29]. The mechanisms underlying the bad vaccine response with aging could be immunosenesis, various anatomical and physiological changes related to aging but also malnutrition and comorbidities; these abnormalities limit the capacity of host vaccine response [29]. The small sample size does not give enough power to statistical tests to identify potential associations between variables of interest. However, the present study provides some guidance and arguments for advocacy to modify the hepatitis B vaccination protocol. Inflammation, hypoalbuminemia and sub-dialysis are very common in chronic hemodialysis patients in Kinshasa; on the other hand, as these factors are associated with a poor vaccine response, it seems more logical to generalize the double dose of HVB vaccine to all patients.

## Conclusion

Half of the patients in the study had either a lack or a low protective vaccine response. Patient-related factors and hemodialysis parameters were the main factors associated with the lack of anti-HbS seroconversion. These results highlight the need to maximize doses of vaccine in all patients.

### What is known about this topic

- Many vaccinations protocols for hepatitis B in hemodialysis patients requires four double dose (40µg) vaccines stretched on a period of 6 months (M0, M1, M2 and M6).

### What this study adds

- It is possible to obtain a positive response to the vaccine (upward of 50%) for patients with CRP < 6 mg/L, albuminemia >30 g/L and KT/V > 1.2, with only a single dose, therefore allowing us to select the class of patients that should use a double dose in the context of high vaccination cost, absence of health insurance and non-subsidized hemodialysis treatment in the DRC.

## Competing interests

The authors declare no competing interests.
